# Visual Behavior Impairments as an Aberrant Sensory Processing in the Mouse Model of Fragile X Syndrome

**DOI:** 10.3389/fnbeh.2019.00228

**Published:** 2019-10-02

**Authors:** Chloé Felgerolle, Betty Hébert, Maryvonne Ardourel, Géraldine Meyer-Dilhet, Arnaud Menuet, Kimberley Pinto-Morais, Jean-Charles Bizot, Jacques Pichon, Sylvain Briault, Olivier Perche

**Affiliations:** ^1^UMR7355, CNRS, Orléans, France; ^2^Experimental and Molecular Immunology and Neurogenetics, University of Orléans, Orléans, France; ^3^Key-Obs, Orléans, France; ^4^Department of Genetics, Regional Hospital, Orléans, France

**Keywords:** Fragile X Syndrome, *Fmr1*^−/y^ mice, FMRP, sensory sensitivity, visual abilities, depth perception, contrast sensitivity

## Abstract

Fragile X Syndrome (FXS), the most common inherited form of human intellectual disability (ID) associated with autistic-like behaviors, is characterized by dys-sensitivity to sensory stimuli, especially vision. In the absence of Fragile Mental Retardation Protein (FMRP), both retinal and cerebral structures of the visual pathway are impaired, suggesting that perception and integration of visual stimuli are altered. However, behavioral consequences of these defects remain unknown. In this study, we used male *Fmr1*^−/y^ mice to further define visual disturbances from a behavioral perspective by focusing on three traits characterizing visual modality: perception of depth, contrasts and movements. We performed specific tests (Optomotor Drum, Visual Cliff) to evaluate these visual modalities, their evolution from youth to adulthood, and to assess their involvement in a cognitive task. We show that *Fmr1*^−/y^ mice exhibit alteration in their visual skills, displaying impaired perspective perception, a drop in their ability to understand a moving contrasted pattern, and a defect in contrasts discrimination. Interestingly, *Fmr1*^−/y^ phenotypes remain stable over time from adolescence to late adulthood. Besides, we report that color and shape are meaningful for the achievement of a cognitive test involving object recognition. Altogether, these results underline the significance of visual behavior alterations in FXS conditions and relevance of assessing visual skills in neuropsychiatric models before performing behavioral tasks, such as cognitive assessments, that involve visual discrimination.

## Introduction

Fragile X Syndrome (FXS) is the most common inherited form of human intellectual disability (ID) affecting approximately 1 in 4,000 males (Penagarikano et al., 2007; Abrahams and Geschwind, 2008; Hunter et al., 2014). This X-linked disorder is characterized by moderate to severe mental retardation, autistic-like behavior, facial abnormalities and macroorchidism (Penagarikano et al., 2007; Hagerman et al., 2017). FXS is caused by the absence of Fragile X Mental Retardation Protein (FMRP) due to transcriptional silencing of the *Fragile X Mental Retardation 1 (*FMR1*)* gene. This FMRP defect leads to numerous synaptic protein alterations (Liao et al., 2008; Klemmer et al., 2011), neuronal dendrite spine immaturity and brain synaptic impairments (Vanderklish and Edelman, 2005; Liao et al., 2008) and thus to cognitive, communication, social and behavioral impairments (Pietropaolo et al., 2011). Besides, FXS patients also present abnormal sensory processing named sensory hypersensitivity (Minshew et al., 1997; Baron-Cohen et al., 2009), characterized by early life strong aversion for visual social contact (over 90% of FXS children), tactile contact or increased noise sensitivity (Lachiewicz et al., 1994; Merenstein et al., 1996).

Among sensory impairments, vision seems particularly affected in FXS patients. Some studies suggested that they provide spatiotemporal visual processing alterations, such as reduced contrast sensitivity for visual stimuli presented at high temporal frequencies as well as altered visual sensitivity for both static (texture difference) and moving stimuli (Kogan et al., 2004b; Farzin et al., 2008). These deficits used to be associated with brain neuronal impairments, in particular in the primary visual cortex, the integrative part of visual system (Kogan et al., 2004a; Farzin et al., 2011). However, we have previously demonstrated that in physiological conditions FMRP is also expressed in the retina, the first structure responsible for light perception (Rossignol et al., 2014; Perche et al., 2018). Subsequently, it has been demonstrated that FMRP is a crucial protein involved in the retinal light sensory (Guimarães-Souza et al., 2016; Wang et al., 2017). Accordingly, using the validated murine FXS model employing *Fmr1*^−/y^ mouse strain (Bakker et al., 1994), we have shown that FMRP deficiency in the retina generates significant abnormalities in proteins contents and cellular alterations leading to defected signal transmission between photoreceptor cells and the inner retina, measured by the electroretinogram (ERG) technique (Rossignol et al., 2014; Perche et al., 2018). Moreover, FMRP absence leads to several deficits in visual subcircuits of the Superior Colliculus (SC; Kay et al., 2018), a midbrain structure regulating eye and head movements. Therefore, absence of FMRP seems to lead to a global visual system defect starting from light perception by the neural retina to visual information integration in cerebral visual areas.

Furthermore, it has been shown that *Fmr1*^−/y^ mouse strain exhibits sensorial impairments, in addition to behavioral and cognitive alterations similar to those observed in FXS patients (Bakker et al., 1994; Nimchinsky et al., 2001; Yan et al., 2004; Dolen et al., 2007; Spencer et al., 2011; Hebert et al., 2014; Ghilan et al., 2018), concomitantly displaying auditory, olfactory and tactile disorders (Larson et al., 2008; Arnett et al., 2014; Rotschafer and Cramer, 2017). However, to our knowledge, the visual skills of *Fmr1*^−/y^ mice have not been investigated using specific visual tests yet. Therefore, we here aimed to study visual aspects of sensory disorders occurring in *Fmr1*^−/y^ mouse strain, especially knowing molecular, cellular and functional defects in *Fmr1*^−/y^ visual system. Of note, vision is an important sense for mice, bringing crucial information for environmental and social understanding (Hoy et al., 2016). Consequently, the visual modality is particularly important when performing behavioral tests in mice. For instance, learning and memorization tests, but also sociability, repetitive behavior or anxiety tests must involve visuospatial skills. It is, therefore, necessary to evaluate the contribution of visual modality on the results obtained during behavioral tests, especially since many of the reported *Fmr1*^−/y^ mouse strain behavioral phenotype were described in test involving visual modality.

Our project aimed to better characterize visual disturbances in *Fmr1*^−/y^ mice from a behavioral point of view. We focused on three traits that characterize visual modality: the perception of contrasts, movements, and depth. Specific tests were carried out in order to carry out specific tests to evaluate visual modalities (Optomotor Drum, Visual Cliff), their evolution from youth to adulthood, and to assess their involvement in a cognitive task.

## Materials and Methods

### Animals

*Fmr1*^−/y^ and their wild-type (WT) males littermates were generated by breeding heterozygous *Fmr1*^–/+^ females from C57BL/6J background with C57BL/6J males. Mice were weaned at 21 days of age and co-housed with their same-sex littermates. Genotype was determined as previously described (Bakker et al., 1994). Food and water were provided *ad libitum*. Animals were maintained under controlled temperature (22°C) and humidity (55%) conditions with a 12:12 h dim light–dark cycle (50 lux, lights on at 7 a.m.). All experimental protocols received full review and approval by the regional animal care and use committee (Comité Régional d’Ethique à l’Expérimentation Animale—CREEA—TSA-DM Therapie1100) prior to conducting the experiments.

### Experimental Design

Investigation of *Fmr1*^−/y^ mice visual phenotype was focused on two main aspects: the depth perception, using the Visual Cliff test, and the contrast and motion understanding, using the Optomotor Drum device. This examination was carried out at three different ages, from adolescence to adulthood, to describe this behavioral phenotype, but also its age of onset and its potential evolution through ages in the absence of FMRP. Therefore, male mice were tested at different ages (1 month, 3 months and 6 months old, respectively), representing adolescence, maturity and the end of mature adulthood. Two groups of animals were used for the Visual Cliff test (WT *n* = 25, *Fmr1*^−/y^ *n* = 22 for 3 months old, WT *n* = 20, *Fmr1*^−/y^ *n* = 26 for 6 months old), and three groups for Optomotor Drum test (WT *n* = 12, *Fmr1*^−/y^ *n* = 18 for 1 month old, WT *n* = 15, *Fmr1*^−/y^ *n* = 13 for 3 months old and WT *n* = 14, *Fmr1*^−/y^ *n* = 14 for 6 months old). In addition, we sharpened our understanding of contrast discrimination using a contrast-shaded Optomotor test (3 months old WT *n* = 48, *Fmr1*^−/y^ *n* = 42). Each animal performed only one contrast condition (lambda: WT *n* = 16, *Fmr1*^−/y^ *n* = 10; beta: WT *n* = 7, *Fmr1*^−/y^ *n* = 6; gamma: WT *n* = 10, *Fmr1*^−/y^ *n* = 14; omega: WT *n* = 6, *Fmr1*^−/y^ *n* = 7; psi: WT *n* = 9, *Fmr1*^−/y^ *n* = 6). Eventually, the implication of visual abilities in a cognitive task involving object recognition was investigated. Three months old mice underwent the Novel Object Recognition (NOR) test and a modified device (WT *n* = 9, *Fmr1*^−/y^ *n* = 13). These two last tests were performed only at one age on the basis of previous results from two first tests.

### Optomotor Drum Visual Test

**Figure 1:**
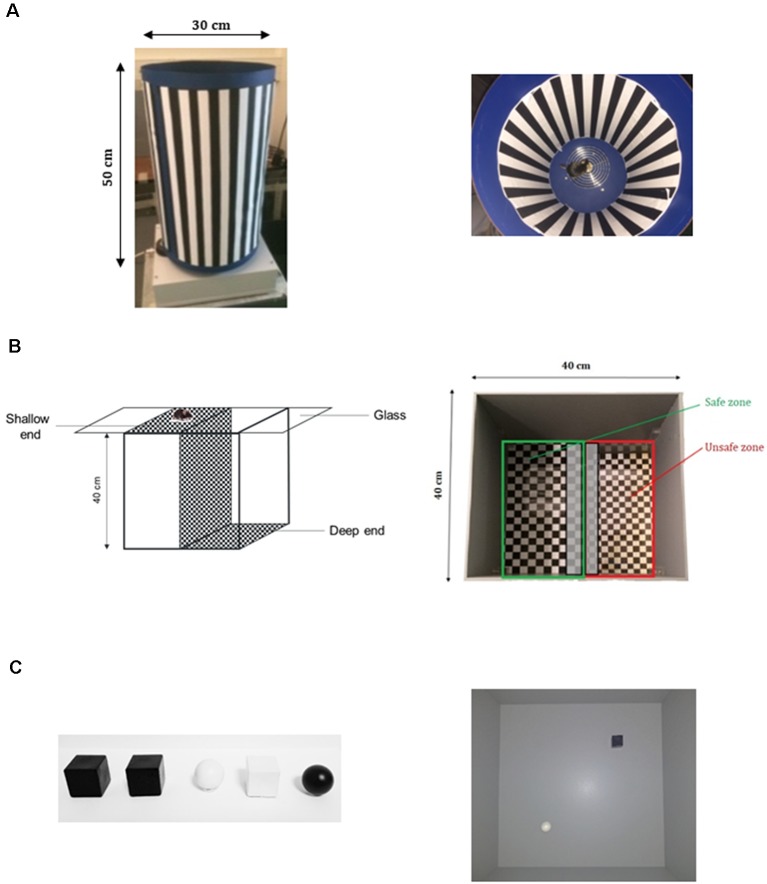
Apparatuses used to perform behavioral tests. **(A)** Optomotor Drum device allowing to provide tested mouse with a contrasted moving pattern, in order to assess contrast perception and contrast discrimination. **(B)** Visual Cliff test assembly composed of a shallow end and a deep end, in order to assess depth perception. **(C)** Novel Object Recognition (NOR) test. On the left, the different objects used as familiar and novel, and on the right the Open field provided with pairs of objects to test the New Object Recognition.

Vision of contrast and motion was assessed with an Optomotor Drum apparatus as previously described (Cowey and Franzini, 1979; Benkner et al., 2013; Kretschmer et al., 2013) with slight modifications. The device is a large rotating circular drum (Ø 30 cm; height 50 cm; Figure 1A). Rotation (constant velocity of 12°/s) was controlled thanks to an electric motor allowing to change the direction of rotation by reversing voltage polarity. In the middle of the cylinder, an elevated stationary metallic platform (20 cm above the drum bottom; diameter: 12 cm) was used to place the tested mouse. The inner side of the drum was covered with a vertical pattern composed of 2 cm-wide black and white stripes, printed on removable paper panels fitting with the cylinder. Homogenous luminance in the apparatus was controlled by lux meter at the height of mouse eyes and was set at 10 lux.

When testing, mouse was placed on the central platform (Figure 1A) and the drum remained motionless during the first 2 min. Then there was a clockwise turn for 2 min, followed by a counterclockwise turn for 2 min. Reversion was immediate to prevent mouse from habituation of the stimulus motion. Recording was also performed when the drum was not moving allowing to check that the mouse did not show spontaneous head movements which may be confounded with head-tracking (HT) movements recorded during test. Habituation to the apparatus without stripes on the drum was performed 24 h before the test. Apparatus was cleaned with 70% ethanol and water between each mouse. All tests were carried out by a single operator blind to mice genotypes.

Videos were recorded by an appropriate video software (EthoVision XT, Noldus, Netherlands), and analyzed by an operator using a video processing software (TheObserver, Noldus, Netherlands). Number and total time duration of HT were counted for each direction of rotation, as well as the latency of the first HT. A single operator, blind to mice genotypes, conducted all analysis.

### Contrast-Shaded Optomotor Drum Test

We investigate *Fmr1*^−/y^ mice contrast sensitivity in order to sharpen our understanding of their vision of contrasts. Contrast sensitivity is the property of vision that measures a local difference in luminance necessary to detect a target. In fact, luminance is the physical value linked to the visual sensation of lightness emitted by a surface. It is defined as the power of a visible light emitted in a surface spot and in a particular direction, expressed by unit of area. Thus, in identical conditions of luminosity, a very light surface would have a high luminance whereas a perfectly black surface would have a null luminance.

To investigate mice behavioral responses toward various contrasted conditions, we created an alternative test using the concept of the Optomotor Drum. The same apparatus was used in similar conditions as described previously.

**Figure 2:**
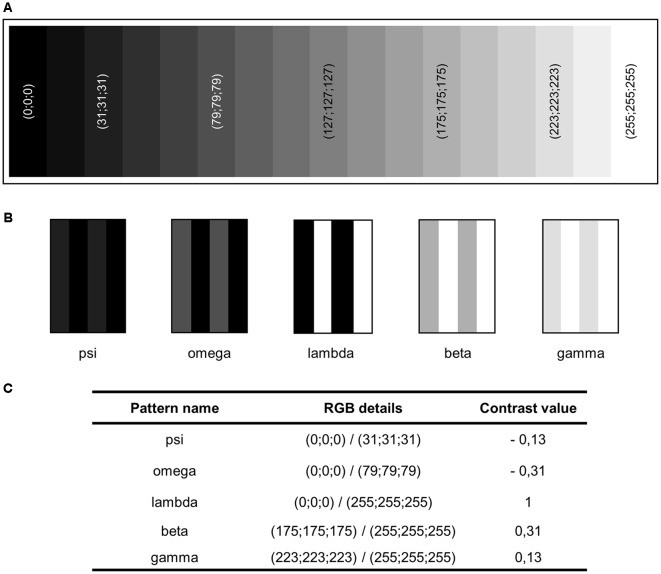
Creation of contrast-shaded patterns used for an adapted Optomotor Drum. **(A)** Gray scale created using the Red Green Blue color value (RGB) color system, from black (0;0;0) to white (255;255;255). **(B)** Contrasted-shaded patterns newly created. **(C)** RGB details of grays used to compose them and contrast value associated (see “Materials and Methods” section for details on contrast computation).

The only difference was in the pattern provided to the tested mouse. Herein, instead of a maximal contrasted pattern (black and white pattern classically used), vertical stripes were composed of a less contrasted pattern, creating shades of gray. We chose to quantify the “gray level,” and so the contrast created, using the Red Green Blue color value (RGB) color system. Briefly, this system constructs all colors as a combination of Red, Green and Blue, and allows to “express” them as three numbers, with values from 0 to 255. Typically, white is (255;255;255) and black is (0;0;0). In the RGB system, grays are expressed with the three same figures in their details. So, the advantage of using the RGB system is that grays are easily expressed. Therefore, we utilized this color system to design a gray scale (Figure 2A) and have chosen five shades of gray to composed new contrast-shaded patterns. Each pattern was composed of 2 cm wide vertical stripes alternating one of the chosen grays and black or white (Figure 2B). Therefore, in our conditions, contrast may be defined as the luminance difference between the two shades of gray used to compose a pattern, since a dark gray has a weak luminance and a light gray has a high luminance. This definition is similar to the one previously used in a study of contrast sensitivity using Optomotor Drum where the authors defined contrast as the difference in luminance between peak and valley of a sine-wave pattern (Umino and Solessio, 2013). We quantified the level of contrast of each pattern by calculating the difference between RGB numbers of the two colors of its stripes and the result was divided by 255 (e.g., if we used (x;x;x) and (y;y;y) with x < y, the contrast was C = (y − x)/255). By convention, contrast was negative when, as compared to the standard condition (lambda), black was conserved and white had been darkened. Following these rules, we composed four contrast-shaded patterns, named beta, gamma, omega and psi (Figure 2C). Gray colors used were printed in a controlled manner, allowing us to perfectly monitor the contrast of the newly created pattern. These contrasted-pattern tests were processed and analyzed exactly as the standard Optomotor Drum test.

### Visual Cliff Test

Depth perception was assessed by Visual Cliff test (Figure 1B) as previously described (Fox, 1965; Glynn et al., 2003), with slight modifications. The apparatus consisted of three plastic boxes: two boxes (length: 20 cm; width: 40 cm; height: 40 cm) were placed side by side on the floor. The first box had its open side directed upward, and a checkboard pattern with black and white squares (2 cm × 2 cm) was settled on its bottom (at the ceiling of the box). The second box had its open side directed downward, and the checkboard pattern was placed on its top. The whole device was covered with a Plexiglas panel. The third box, bottomless and roofless (length: 40 cm; width: 40 cm; height: 40 cm) was placed above. In this setting, the third box represented an open field composed of two equal sections: the section above the first box (length: 20 cm; width: 40 cm) with a cliff of 40 cm discernable by the tested mouse between the Plexiglas panel and the checkerboard-patterned bottom, called “deep end” (or “unsafe zone,” red zone on the figure), and the section above the second box (length: 20 cm; width: 40 cm) called “shallow end” (or “safe zone,” green zone on the figure; Figure 1B). Homogenous luminance in the apparatus was controlled by lux meter at the height of mouse eyes and was set at 10 lux.

During testing, a mouse was placed in the corner of the shallow end section and was free to move all over the open field for 300 s. Its behavior was recorded by a video camera through appropriate video software (EthoVision XT10, Noldus, Netherlands). Apparatus was cleaned with 70% ethanol and water between each mouse. A single operator blind to mouse genotypes conducted all assessment.

Videos were analyzed by video-tracking software (EthoVision XT10, Noldus, Netherlands). For the analysis, the open field was separated in two distinct zones: safe and unsafe zones.

Time spent in each zone was counted, and Preference Index (PI) for the safe zone [PI = (Time in safe zone/(Total test time) × 100)] was calculated for each mouse. Occurrences in each zone, as well as the total number of crossings between the two zones were also quantified. The total distance moved by the mouse during the test was also recorded.

### Novel Object Recognition Task (NOR)

The NOR task is a cognitive test about visual recognition memory. It is based on mouse innate tendency to preferentially explore novel stimuli, and allows to assess memory without external motivation, rewards or punishment (Ennaceur and Delacour, 1988) by measuring an index of stimulus recognition (Baxter, 2010). Briefly, the standard task (NOR1) consisted of three phases: habituation, familiarization, and choice (Bhattacharya et al., 2012; King and Jope, 2013; Gomis-González et al., 2016; Costa et al., 2018). The mouse was habituated to an empty open-field (40^*^40^*^40 cm) for 10 min, 48 h and 24 h before the task. This habituation (Phase 1) reduces mouse anxiety linked to the confrontation to a novel environment. The test day, the mouse was first given 3 min to rehabituate to the empty arena, then a pair of identical objects (black plastic cube; 3^*^3^*^3 cm) was placed in diagonally opposite corners of the arena, approximately 5 cm from the walls of the box to allow investigation of all sides of the objects (Figure 1C). The mouse was then placed in the unoccupied corner facing the wall and allowed to freely investigate the two objects for 5 min (Phase 2 or familiarization). During the Phase 3 (or choice), objects were substituted by an identical object to the previous ones and a novel object different in color and shape (white plastic ball; Ø3 cm; Figure 1C). Both objects were changed in order to exclude the contribution of the sense of smell in the object recognition performed by the tested mouse. Once again, the mouse was allowed to freely investigate the two objects during 5 min. These two phases were separated by a 3-min intertrial interval (ITI). The mouse was removed from the open-field and placed in a single-housing cage next to the arena for the duration of the ITI. We alternated the location of the novel object, so that half of the mice saw the novel object on their left and the other half saw it on their right. After each session, the open-field and objects were wiped with 70% ethanol to eliminate olfactory cues. All tests were carried out at 20 lux luminance by a single operator blind to mice genotypes.

In order to investigate how color and shape impact the recognition of novel object, we generated two slightly modified versions of the NOR task, named NOR2 (color-modified version) and NOR3 (shape-modified version). The difference among the three versions relies on the presented objects during the choice phase: during the NOR2 the novel object differed from the familiar by its color (white plastic cube; 3^*^3^*^3 cm) and during the NOR3 the novel object differed by its shape (black plastic ball; Ø3 cm; Figure 1C).

This object recognition test was set up with black and white objects in order to draw a parallel with the Optomotor Drum test. The idea was to use a contrast between the two objects of the same intensity that black and white stripes of the Optomotor Drum. The texture characteristic of the object was deliberately retained in order to modify only visual characteristics without interfering with the tactile modality.

Videos were analyzed by video-tracking software (EthoVision XT10, Noldus, Netherlands). Object exploration was scored only when the mouse’s nose or front paws were in contact with the object. Duration (time) and frequency (number of visits) of object exploration during each phase were recorded, and a discrimination index representing the difference of time exploring each object between the familiarization phase and the choice phase (expressed in percentage of the exploration time during familiarization phase).

### Statistical Analysis

All results are expressed as mean ± SEM. Data analysis was performed using Statistica 13.3. For all statistical tests, a confidence interval of 0.95 was initially chosen (i.e., *α* = 0.05). For all data, we tested the distributions’ normality with the Shapiro–Wilk’s test, and the homogeneity of variances with Levene’s test when an analysis of variance (ANOVA) analyze was required. For Visual Cliff and NOR tests, we used repeated-measures ANOVA followed by *post hoc* Fisher’s LSD test when a statistically significant main effect or interaction was detected (*p* < 0.05). For the Visual Cliff test, main factors were zone (Fz), genotype (Fg) and age (Fa). For NOR tests main factors were object (Fo) and genotype (Fg). For Optomotor tests, statistical analysis was conducted using two-way ANOVA with age (Fa), genotype (Fg), or a three-way ANOVA with age (Fa), genotype (Fg) and minute (Fm) as main factors. For contrast-shaded Optomotor, we used a repeated-measures ANOVA followed by *post hoc* Fisher’s LSD test with contrast condition (Fc) and genotype (Fg) as main factors. For all tests, when non-parametric tests were required, a Kruskal–Wallis test was realized followed by Wilcoxon’s test with a *p* correction. In figures, significant differences between groups are noted by ^*^p < 0.05; ^**^p < 0.01; ^***^p < 0.001; ^****^p < 0.0001 for genotype comparisons, and by ^#^*p* < 0.05; ^##^*p* < 0.01; ^###^*p* < 0.001; ^####^*p* < 0.0001 for intra-groups comparisons.

## Results

### Contrast Perception Impairment in *Fmr1*^−/y^ Mice

Motion and contrast understanding of *Fmr1*^−/y^ mice were investigated thanks to the Optomotor Drum. This apparatus is based on a natural reflex: a mouse with visual abilities efficient enough to detect a contrasted motion passing through its visual field would have the reflex to follow this stimulus with its head, at least for a brief moment (Mitchiner et al., 1976; Schmucker et al., 2005; Kretschmer et al., 2015). This instinctive movement, called the optomotor response and illustrated in practice by a HT, reflects the ability to detect and understand a contrasted motion occurring in the visual field, and so is not due to cognitive and computational abilities. This test rests on the optokinetic nystagmus reflex occurring when a visual target moves across the visual field (Mitchiner et al., 1976), reflecting the animal perception of a moving stimulus. Since for rodents the optomotor response is well correlated to the optokinetic reflex (Kretschmer et al., 2017), the Optomotor Drum test has been described to be a robust test to investigate vision of contrasts abilities in mice, allowing data collection without invasive procedure (Thaung et al., 2002). Therefore, this test performed without any motion limitation allows to gain information on the visual abilities without training, exacerbated anxiety, and overall without involvement of mouse cognitive abilities, and has been demonstrated to be a very robust test to discriminate rodents with normal vision from those with visual alterations (Lawrence et al., 2000; Schmucker et al., 2005; Akimov and Rentería, 2012; Umino and Solessio, 2013).

**Figure 3:**
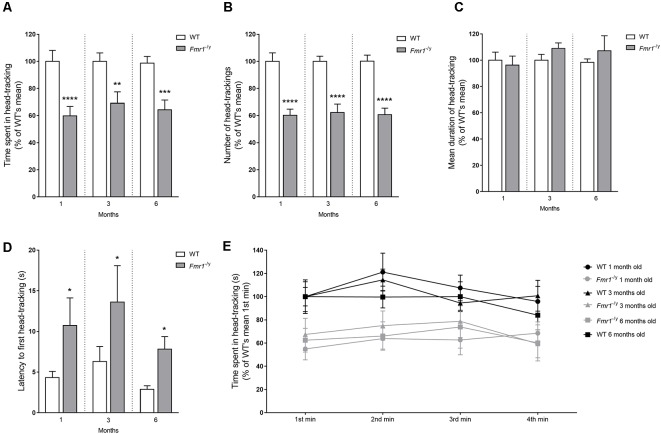
Optomotor Drum test. Motion and contrast perception of *Fmr1*^−/y^ mice were investigated thanks to the Optomotor Drum. Histograms represent **(A)** total time spent in head-tracking (HT), **(B)** total number of HT, **(C)** mean duration of one HT and **(D)** latency to the first HT. Curve represents **(E)** time spent in HT minute by minute. All data were represent at each age tested (1 month: wild-type (WT) *n* = 12; *Fmr1*^−/y^ *n* = 18; 3 months: WT *n* = 15; *Fmr1*^−/y^ *n* = 13; 6 months: WT *n* = 14; *Fmr1*^−/y^ *n* = 14). All parameters were scored in seconds, and were expressed in % of results obtained with the WT group at the corresponding age (WT littermates). Data represent mean ± SEM. Significant differences between WT and *Fmr1*^−/y^ are noted by ^*^p < 0.05; ^**^p < 0.01; ^***^p < 0.001; ^****^p < 0.0001.

This test was carried out on 1-3- and 6-month-old mice. All mice were able to perform HT, and the operator did not observe any difference in the way HT were performed between genotypes. Parameters recorded were the number of HT and the total time spent in HT, from which were calculated the mean duration of a HT (Figure 3). For all these parameters, statistical analysis did not revealed any interaction between age and genotype (duration *F*_a,g(2,80)_ = 0.221, *p* = 0.802; number *F*_a,g(2,80)_ = 0.022, *p* = 0.978; mean duration WT $\chi_{\left(2\right)}^{2}$ = 0.295, *p* = 0.863; *Fmr1*^−/y^ $\chi_{\left(2\right)}^{2}$ = 2.93, *p* = 0.230). However, a genotype effect on both total time spent in HT (*F*_g(1,80)_ = 37.53, *p* < 0.0001) and the number of HT (*F*_g(1,80)_ = 90.84, *p* < 0.0001) was highlighted. Indeed, *Fmr1*^−/y^ mice spent significantly less total time in HT as compared to their WT littermates (1 month: *p* = 0.0001; 3 months: *p* = 0.002; 6 months: *p* = 0.0008; Figure 3A), resulting from a significant decrease in number of HT (1 month: *p* < 0.0001; 3 months: *p* < 0.0001; 6 months: *p* < 0.0001; Figure 3B). *Fmr1*^−/y^ mice provided a 40% decreased response as compared to WT ones (Figures 3A,B). Importantly, there was no genotype effect on the mean duration of an HT. Indeed, no significant difference was obtained for this parameter between WT and *Fmr1*^−/y^ mice (1 month: *p* = 0.433; 3 months: *p* = 0.140; 6 months: *p* = 0.908; Figure 3C), highlighting that variation observed in the total time spent in HT was directly linked to the number of HT, and not to a variation in the duration of each HT. This underlines that the ability to perform a correct HT was not altered in *Fmr1*^−/y^ mice and that their head and neck motion did not hinder their task performance. Moreover, similar results were obtained when considering each rotation direction (clockwise and counterclockwise), whatever the age (Supplementary Figure S1).

Ultimately, it is important to highlight that decreased total duration and number of HT observed in *Fmr1*^−/y^ mice were similar across all three tested ages, approximately 35%, from 1 to 6 months old.

We recorded the latency to the first HT provided by each mouse, meaning the time between the first HT movement and the start of the drum rotation. Statistical analysis did not reveal any significant difference between results at 1, 3 and 6 months, whatever the genotype (WT $\chi_{\left(2\right)}^{2}$ = 0.806, *p* = 0.668; *Fmr1*^−/y^ $\chi_{\left(2\right)}^{2}$ = 1.083, *p* = 0.582). One-month-old *Fmr1*^−/y^ mice showed a significant (*p* = 0.015) increase in latency to the first HT in regards to WT. Results remained stable from one age to another (Figure 3D), with a significant difference between WT and *Fmr1*^−/y^ mice in their latency to first HT (3 months: *p* = 0.016; 6 months: *p* = 0.013). These results underline the lower ability of *Fmr1*^−/y^ mice in contrast perception. Moreover, we quantified the time spent in HT at each minute of the test (Figure 3E). In accordance with previous results, statistical analysis showed a genotype effect on this parameter (*F*_g(1,80)_ = 0.699, *p* < 0.0001) without any interaction between age and genotype (*F*_a,g(2,80)_ = 0.299, *p* = 0.742). Moreover, no effect of the minute of the test was noticed (*F*_m(3,80)_ = 2.31, *p* = 0.076) without any significant interaction between genotype and the minute of the test (*F*_g,m(3,80)_ = 0.777, *p* = 0.507). These results indicate that for all three tested ages WT and *Fmr1*^−/y^ mice spent similar time in HT at each minute of the test. These data show that the response to this test remained stable throughout the whole test duration, whatever the genotype. Thus, the overall decreased response of *Fmr1*^−/y^ mice was not due to shorter HT, or to a decrease occurring during the test. *Fmr1*^−/y^ mice had a similar mean duration of HT than WT, and their response remained stable during the 4 min of the test, whatever the rotation direction of the drum.

*Fmr1*^−/y^ mice spent less time in HT than WT ones, due to a reduced number of HT. This decrease was not due to disinterest or a lack of attention span that occurred when mice performed an HT, and throughout the whole test duration. As illustrated by their clearly raised latency to the first HT, *Fmr1*^−/y^ mice showed difficulties in the detection of the contrasted stimulus.

Globally, the lower response of *Fmr1*^−/y^ mice to the Optomotor Drum test highlighted defects in vision of contrast and motion, from youth to adulthood, in a stable way through the ages.

### Contrast Discrimination Alterations in *Fmr1*^−/y^ Mice

In order to sharpen our understanding of the defects in vision of contrasts of *Fmr1*^−/y^ mice, we reused the Optomotor Drum to investigate their behavioral response toward various contrasted conditions. The standard test showed that mouse responses remained stable from 1 to 6 months old, whatever the genotype. Therefore, seeking for a refinement of animals used, the Contrast-shaded Optomotor Drum test was performed only at one age (3 months) for all conditions tested. As for the standard test, parameters recorded were the time spent in HT and the number of HT, from which the mean duration of a single HT was deduced.

**Figure 4:**
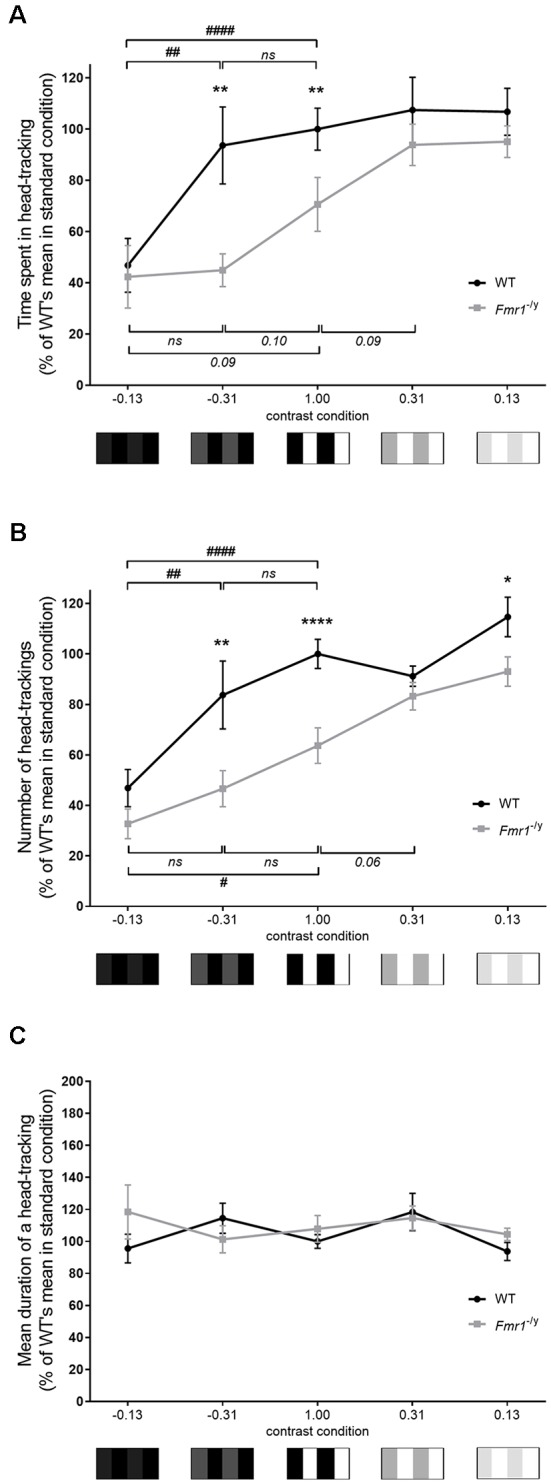
Contrast-shaded Optomotor Drum test. Contrast discrimination alterations of *Fmr1*^−/y^ mice were investigated thanks to the Optomotor Drum, with various contrasted conditions. Curve representing **(A)** total time spent in HT and **(B)** total number of HT, hence **(C)** the mean duration of one HT, at each contrast condition (lambda: WT *n* = 16, *Fmr1*^−/y^ *n* = 10; beta: WT *n* = 7, *Fmr1*^−/y^ *n* = 6; gamma: WT *n* = 10, *Fmr1*^−/y^ *n* = 14; omega: WT *n* = 6, *Fmr1*^−/y^ *n* = 7; psi: WT *n* = 9, *Fmr1*^−/y^ *n* = 6). Total durations and mean durations scored in seconds. Total durations and number expressed in % of result of WT mice in lambda condition (standard condition). Data represent mean ± SEM. Significant differences between WT and *Fmr1*^−/y^ are noted by ^*^p < 0.05; ^**^p < 0.01; ^****^p < 0.0001. Significant differences between contrast conditions within genotype are noted by ^#^*p* < 0.05; ^##^*p* < 0.01; ^####^*p* < 0.0001. Absence of significant difference between groups is noted by ns.

First, it has to be noted that whatever the genotype mice responded to all contrast conditions tested. These conditions did not allow to determine a threshold beyond which contrast would be too weak to be perceived (Figure 4).

Then, irrespectively of the genotype, the observed response depended on the contrast used. Indeed, statistical analysis showed an effect of contrast condition (*F*_c(4,81)_ = 10.15, *p* < 0.0001), but also a genotype effect (*F*_g(1,81)_ = 11.49, *p* = 0.001) on the total time spent in HT (Figure 4A). The evolution of time spent in HT, in regards to contrasts, differed between genotypes. Indeed, at *C* = −0.31 WT mice spent significantly more time in HT than at *C* = −0.13 (*p* = 0.003), reaching a similar duration to the one performed at *C* = 1 (*p* = 0.653). In contrast, at *C* = −0.31 *Fmr1*^−/y^ mice spent a similar amount of time in HT to the one performed at *C* = −0.13 (*p* = 0.874) and a very low amount of time compared to the one recorded in standard condition (*p* = 0.098). Consequently, even if the time spent in HT was similar between both genotypes at *C* = −0.13 (*p* = 0.772), it became significantly different as contrast reached −0.31 (*p* = 0.003) and remained different when *C* = 1 (*p* = 0.008), as previously described with the standard Optomotor Drum. Thus, from *C* = −0.13 to *C* = 1, the time spent in HT increased whatever the genotype, but following different patterns for each genotype. Interestingly, mice did not provide these evolutions when contrast varied from 0.13 to 1. Compared to standard condition, WT mice spent a similar time in HT at *C* = 0.31 (*p* = 0.577) and at *C* = 0.13 (*p* = 0.569). Thus, WT mice did not decrease their time spent in HT when contrast was reduced. On the contrary, *Fmr1*^−/y^ mice spent more time in HT at *C* = 0.31 than at 1 (*p* = 0.089), but not at *C* = 0.13 (*p* = 0.931). Thus, *Fmr1*^−/y^ mice increased their time spent in HT and remained stable when contrast was reduced from 1 to 0.13. Consequently, the difference in the time that mice spent in HT between WT and *Fmr1*^−/y^ was not significant anymore at *C* = 0.31 (*p* = 0.409) nor at 0.13 (*p* = 0.341).

In accordance with results obtained with the standard test, whatever the genotype, the time spent in HT was correlated to the number of HT (Figure 4B). Results regarding the number of HT followed identical trends than those described for total duration spent in HT. Indeed, statistical analysis highlighted an impact of contrast (*F*_c(4,81)_ = 20.98, *p* < 0.0001) and of genotype (*F*_g(1,81)_ = 24.15, *p* < 0.0001) on number of HT performed by mice. Whatever the genotype number of HT varied with the contrast tested, and the evolution of the number of HT as a function of the contrast differed between genotypes. At a contrast of −0.31 WT mice performed more HT than at −0.13 (*p* = 0.0019), reaching a score similar to the one performed at *C* = 1 (*p* = 0.123), whereas, *Fmr1*^−/y^ mice performed a number of HT similar to the one at −0.13 (*p* = 0.255) and lower from the standard condition score (*p* = 0.161). Therefore, even if the number of HT was similar between genotypes at *C* = −0.13 (*p* = 0.223), it became different as the contrast reached −0.31 (*p* = 0.003) and remained different in standard condition (*p* < 0.0001). Thus, when the contrast increased from −0.13 to 1, the number of HT increased for both genotypes, but at different rates for each genotype. As for the time spent in HT, evolutions of the number of HT were different when contrast varied from 0.13 to 1. In regards to standard condition, WT mice performed a similar number of HT at *C* = 0.31 (*p* = 0.375) and at *C* = 0.13 (*p* = 0.100). Then WT mice did not decrease their number of HT when contrast was reduced. On the contrary, *Fmr1*^−/y^ mice performed a higher number of HT at *C* = 0.31 than at *C* = 1 (*p* = 0.060) but not at *C* = 0.13 (*p* = 0.361). Thus, *Fmr1*^−/y^ mice increased their number of HT and then remained stable when contrast decreased from 1 to 0.13. Based on these results, the mean duration of a HT was calculated. It remained similar between genotypes whatever the contrast condition (Figure 4C); thus, results previously obtained were not due to a difference in the duration of an HT between genotypes.

It is interesting to highlight that, irrespective of the genotype, for two patterns with an identical gap in the RGB scale, mice did not provided the same responses. Those depended on whether the contrasted pattern was in light or in dark shades of gray. However, WT and *Fmr1*^−/y^ mice did not provide similar evolution through contrasts, leading to significant differences of response for several contrasts tested. Together, these results show that mice have difficulties distinguishing a sharp contrast in a dark condition. However, as soon as the contrast was somewhat increased WT mice reached their maximal score in contrast detection, whereas *Fmr1*^−/y^ mice did not enhance their response. *Fmr1*^−/y^ mice required a far more increased contrast to enhance their contrast detection ability and leave their basal response. Therefore, *Fmr1*^−/y^ mice display impaired discrimination of contrasts. Results highlighted a shifting in *Fmr1*^−/y^ mice response to increased contrast, with a rise in response postponed on the contrast scale, meaning that *Fmr1*^−/y^ mice have a lower sensitivity to contrast.

### Depth Perception Deficiency in *Fmr1*^−/y^ Mice

We assessed depth perception in *Fmr1*^−/y^ mice by subjecting them to the Visual Cliff test, which has been shown to be effective in distinguishing between animals with normal and poor visual abilities (Fox, 1965). Main parameters recorded were the total occurrences and time spent by mice in each zone (safe, unsafe), but also distance moved. Three and 6-months-old mice underwent this test.

#### Time and Occurencies

**Figure 5:**
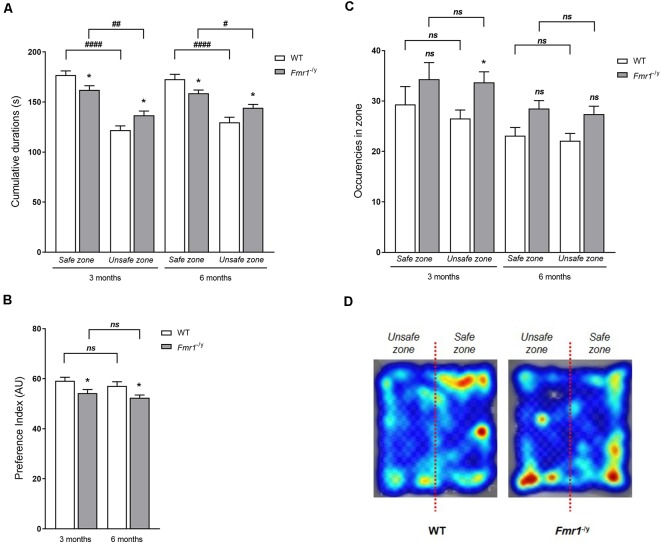
Visual Cliff test. Depth perception in *Fmr1*^−/y^ mice was assessed with the Visual Cliff test. Histograms represent** (A)** total time spent in each zone (shallow-end/deep-end), hence **(B)** the Preference Index (PI) for the shallow end was calculated, and **(C)** number of occurrences, at both ages tested (3 months: WT *n* = 25; *Fmr1*^−/y^ *n* = 22; 6 months: WT *n* = 20; *Fmr1*^−/y^ *n* = 26). **(D)** Illustration by a pseudo-colored heat map representing time spent at each position related to the place preference of WT and *Fmr1*^−/y^ mice. Total durations scored in seconds; index expressed in Arbitrary Unit (AU). Data represent mean ± SEM. Significant differences between WT and *Fmr1*^−/y^ are noted by ^*^p < 0.05. Significant differences between zones within genotype are noted by ^#^*p* < 0.05; ^##^*p* < 0.01; ^####^*p* < 0.0001. Absence of significant difference between groups is noted by ns.

**Figure 6:**
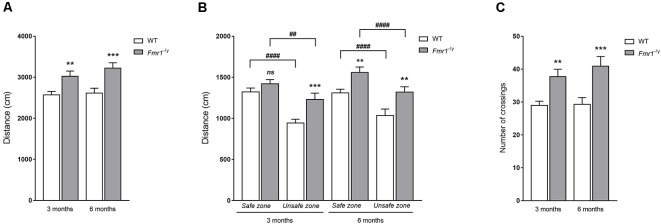
Locomotion parameters in the Visual Cliff test. Locomotion was assessed thanks to total distances moved during the Visual Cliff test in **(A)** the whole arena, **(B)** safe and unsafe zones at both ages tested, and thanks to **(C)** the number of transitions between deep and shallow ends (3 months: WT *n* = 25; *Fmr1*^−/y^ *n* = 22; 6 months: WT *n* = 20; *Fmr1*^−/y^ *n* = 26). Distance scored in centimetre. Data represent mean ± SEM. Significant differences between WT and *Fmr1*^−/y^ are noted by ^**^p < 0.01; ^***^p < 0.001. Significant differences between zones within genotype are noted by ^##^*p* < 0.01; ^####^*p* < 0.0001. Absence of significant difference between groups is noted by ns.

First, age had no impact on occurrences and time in zones or on the PI for the safe zone. Indeed, no significant interaction between zone, genotype and age was noticed (time in zones *F*_z,a,g(1,90)_ = 0.28, *p* = 0.94; PI, *F*_a,g(1,90)_ = 0.002, *p* = 0.96; occurence *F*_z,a,g(1,90)_ = 0.28, *p* = 0.59). Each genotype provided similar behavioral profiles in regard to occurrences and time spent in zones, and so for PI, whatever the age (Figures 6A–C). Moreover, a zone effect (*F*_z(1,90)_ = 67.47, *p* < 0.0001) was observed on the total time spent in each zone (Figure 5A), with more time spent in the safe zone than in the unsafe zone whatever the genotype (3 months: WT *p* < 0.0001; KO *p* = 0.004; 6 months: WT *p* < 0.0001; KO *p* = 0.041). However, a significant interaction between zone and genotype (*F*_g,z(1,90)_ = 12.04, *p* = 0.0008) was noticed. Indeed, as compared to WT mice, *Fmr1*^−/y^ mice spent significantly more time in the unsafe zone (3 months: *p* = 0.014; 6 months: *p* = 0.017) and less time in the safe zone (3 months: *p* = 0.014; 6 months: *p* = 0.019; Figure 5A). Based on these results, a PI for the safe zone was calculated for each mouse. A genotype effect (*F*_g(1,90)_ = 12.02, *p* = 0.0008) was observed on PI since *Fmr1*^−/y^ mice had a significantly lower PI than WT mice (3 months: *p* = 0.013; 6 months: *p* = 0.018). This index suggests that *Fmr1*^−/y^ mice showed a decreased preference for the safe zone compared to WT (Figure 5B). Regarding occurrences in each zone, a genotype effect (*F*_g(1,90)_ = 8.07, *p* = 0.005) appeared on entry frequency in safe and unsafe zones (Figure 5C). Indeed, as compared to WT mice, *Fmr1*^−/y^ mice performed a higher number of occurrences in the safe zone (3 months: *p* = 0.120; 6 months: *p* = 0.097) as in the unsafe zone (3 months: *p* = 0.030; 6 months old: *p* = 0.102). Moreover, the zone had no effect on the number of occurrences (*F*_z(1,90)_ = 1.69, *p* = 0.196). The number of entries in each zone were similar between safe and unsafe zones for WT mice (3 months: *p* = 0.174; 6 months: *p* = 0.655) as for *Fmr1*^−/y^ mice (3 months: *p* = 0.771; 6 months: *p* = 0.592; Figure 5C), explaining why the interaction between genotype and zone was not statistically significant (*F*_g,z(1,90)_ = 0.242, *p* = 0.623). Thus, genotype imbalance observed in times spent in each zone is not due to the number of visits in each zone, as illustrated by the heat map recorded for each genotype (Figure 5D).

Herein, *Fmr1*^−/y^ mice showed an altered behavior in the Visual Cliff characterized by an increased time in unsafe zone without increase in the number of entries in this zone. It is worth noting that whatever the genotype, mice spent more time in the safe zone than in the unsafe zone. Consequently, our results did not show a loss of the preference for the safe zone, but only a decrease in the preference for the safe zone. This imbalance in the presence in each zone led to a clear decrease of the PI for the safe zone of *Fmr1*^−/y^ mice.

#### Locomotion

Regarding locomotion features (Figure 6), a genotype effect (*F*_g(1,90)_ = 19.40, *p* < 0.0001) was observed on the total distance covered during the test without any significant interaction between genotype and age (*F*_g,a(1,90)_ = 0.315, *p* = 0.576). Indeed, 3-month-old *Fmr1*^−/y^ mice demonstrated a significantly higher distance traveled in regards to WT ones (*p* = 0.007), and 6-month-old mice presented a similar profile, with *Fmr1*^−/y^ mice covering a significantly higher distance than WT ones (*p* = 0.0007; Figure 6A). Furthermore, the distance moved in each zone of the apparatus was quantified (Figure 6B). A zone effect (*F*_z(1,90)_ = 80.20, *p* < 0.0001) was noticed, but there was no significant interaction between genotype and zone (*F*_g,z(1,90)_ = 3.79, *p* = 0.054), nor between zone and age (*F*_a,z(1,90)_ = 0.097, *p* = 0.756). These results illustrate that mice covered a significantly shorter distance in a deep zone than in a shallow zone, whatever the genotype and age (3 months: WT *p* < 0.0001; *Fmr1*^−/y^ *p* = 0.003; 6 months: WT *p* < 0.0001; *Fmr1*^−/y^ *p* < 0.0001; Figure 6B). A genotype effect was observed (*F*_g(1,90)_ = 17.90, *p* < 0.0001) on the distance covered in the shallow end as well as in the deep end but statistical analysis did not show a significant interaction between age and genotype (*F*_a,g(1,90)_ = 0.342, *p* = 0.5604). Indeed, 3-month-old *Fmr1*^−/y^ mice walked a higher distance than WT ones in safe zone (*p* = 0.253) as in unsafe zone (*p* = 0.001), and a similar profile was observed at 6 months old, with *Fmr1*^−/y^ mice traveling a significant higher distance than WT mice in the shallow end (*p* = 0.009) as in the deep end (*p* = 0.001; Figure 6B).

Ultimately, the total number of crossings between safe and unsafe zones performed by tested mice was recorded (Figure 6C). A genotype effect (*F*_g(1,90)_ = 19.53, *p* < 0.0001) was obtained without any significant interaction between age and genotype (*F*_g,a(1,90)_ = 0.282, *p* = 0.596), illustrating that *Fmr1*^−/y^ mice performed a significantly greater number of crossings between shallow and deep ends than WT mice at both ages (3 months old: *p* = 0.007; 6 months old: *p* = 0.0007).

Together, these results on locomotion were in line with the well-known hyperactive behavior of *Fmr1*^−/y^ mice as described in the literature (Kramvis et al., 2013; Mines, 2013; Sørensen et al., 2015) in regards the distance moved in the arena. *Fmr1*^−/y^ mice covered a significantly longer distance than WT mice, in the shallow end as well as in the deep end. Therefore, as the hyperactivity occurred homogeneously in every zones of the arena, mouse locomotion did not explain the results we obtained in time and frequencies in zones. Even if *Fmr1*^−/y^ mice covered a longer total distance than WT ones during the test, this increase occurred in safe as in unsafe zone. Imbalance in the time spent in zones cannot be attributed to locomotion activity.

Thus, the imbalanced PI for the shallow end is not linked to locomotion phenotype in the *Fmr1*^−/y^ mice. The impaired response to the Visual Cliff test suggested that *Fmr1*^−/y^ mice had difficulties in depth perception, at 3 and 6 months old.

### Involvement of Visual Modalities Impaired in *Fmr1*^−/y^ Mice in a Cognitive Task

Visual memory recognition was evaluated thanks to the NOR task, in its standard version and in two modified tasks. This test rests on rodents’ innate exploratory behavior when they are exposed to a novel and a familiar objects, and allows to assess memory without external motivation, rewards or punishment (Ennaceur and Delacour, 1988) by measuring an index of stimulus recognition (Baxter, 2010). Previous visual tests showed that mouse responses remained stable from 1 to 6 months old, whatever the genotype. Therefore, seeking for a refinement of animals used, NOR tests were performed only at one age (3 months old mice). For each phase, the number of nose contact with each object and the time spent exploring each object presented were recorded, hence a discrimination index for each object was calculated.

**Figure 7:**
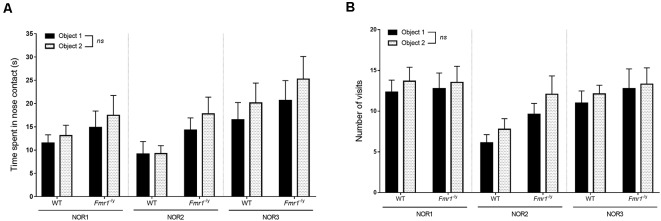
Familiarization phase of the NOR. *Fmr1*^−/y^ behavior in a cognitive task involving visual modality was evaluated in the standard version of the NOR (NOR1) and two various versions (NOR2 and NOR3). Histograms represent **(A)** time spent in nose contact with each object and **(B)** number of visits of each object for each genotype (WT *n* = 9, *Fmr1*^−/y^ *n* = 13), during the familiarization phase. Durations scored in seconds. Data represent mean ± SEM.

For the three versions of the test, the two identical objects presented in the familiarization phase were two black plastic cubes. During this phase, durations spent with objects were not impacted by the object (NOR1 *F*_o(1,20)_ = 0.877, *p* = 0.360; NOR2 *F*_o(1,20)_ = 1.26, *p* = 0.274; NOR3 *F*_o(1,20)_ = 2.01, *p* = 0.170) and did not undergo interaction of the genotype (NOR1 *F*_g,o(1,20)_ = 0.004, *p* = 0.826; NOR2 *F*_g,o(1,20)_ = 1.187, *p* = 0.288; NOR3 *F*_g,o(1,20)_ = 0.025, *p* = 0.873; Figure 7A). Indeed, WT and *Fmr1*^−/y^ mice did not spend more time with one object than with the other. These results were coherent with the number of visits which were not impacted by the object (NOR1 *F*_o(1,20)_ = 0.914, *p* = 0.350; NOR2 *F*_o(1,20)_ = 3.75, *p* = 0.067; NOR3 *F*_o(1,20)_ = 0.241, *p* = 0.628) nor by the genotype (NOR1 *F*_g,o(1,20)_ = 0.065, *p* = 0.800; NOR2 *F*_g,o(1,20)_ = 0.139, *p* = 0.713; NOR3 *F*_g,o(1,20)_ = 0.029, *p* = 0.866; Figure 7B). These results suggested that there was no bias caused by a preference for an object due to its location. Moreover, no difference in global exploration was noticed between WT and *Fmr1*^−/y^ mice since both genotypes spent the same total time exploring objects (NOR1 *F*_g(1,20)_ = 0.74, *p* = 0.40; NOR2 *F*_g(1,20)_ = 3.01, *p* = 0.099; NOR3 *F*_g(1,20)_ = 0.64, *p* = 0.63).

**Figure 8:**
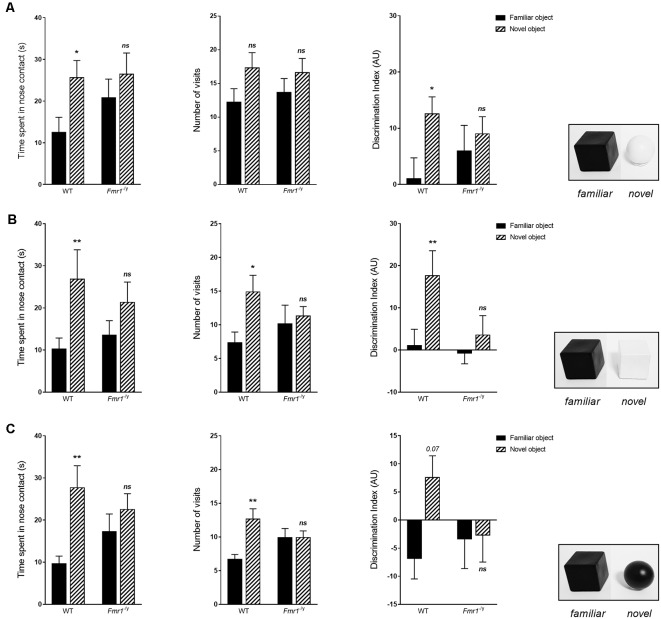
Choice phase of the NOR. Histograms represent time spent in nose contact with each object and number of visits, hence a discrimination index for each object, for **(A)** NOR1, **(B)** NOR2 and **(C)** NOR3, for each genotype (WT *n* = 9, *Fmr1*^−/y^ *n* = 13), during the choice phase. Durations scored in seconds; discrimination index expressed in AU. Data represent mean ± SEM. Significant differences between WT and *Fmr1*^−/y^ are noted by ^*^p < 0.05; ^**^p < 0.01. Absence of significant difference between groups is noted by ns.

After a 3 min ITI, one of the two cubes was replaced by a new object, and the other cube was replaced by a duplicate cube. For the standard version of the test (NOR1), this object (a white plastic ball) differed by its shape and color. During the choice phase, statistical analysis revealed an impact of the object on the time spent in exploration (*F*_o(1,20)_ = 6.875, *p* = 0.016) and on the number of visits (*F*_o(1,20)_ = 5.858, *p* = 0.025). Indeed, WT mice spent significantly more time in contact with the new object (*p* = 0.027), and tended to visit the new object more (*p* = 0.058), whereas *Fmr1*^−/y^ mice did not present difference in their exploration of the two objects (time: *p* = 0.232; number: *p* = 0.183; Figure 8A). We obtained results similar to the literature (Bhattacharya et al., 2012; King and Jope, 2013; Gomis-González et al., 2016; Costa et al., 2018), with WT mice spending more time exploring the new object and *Fmr1*^−/y^ mice spending as much time sniffing the familiar object and the new one during the choice phase, although the total time of exploration of objects was similar between both genotypes. The frequency of approach to objects corroborated this result, since WT mice more often visited the new object than the familiar one, whereas *Fmr1*^−/y^ mice explored them similarly. This phenomenon is also represented by the discrimination index which corresponds to the difference of time spent exploring objects between the familiarization phase and the choice phase. Indeed, the discrimination index indicates that the time spent by WT mice sniffing the new object was significantly greater than the time spent in contact with the object replaced during the familiarization phase, even if WT mice explored the unchanged cube in the same proportion. Thus, WT mice have a significantly higher index for the new object vs. the familiar object (*p* = 0.052), whereas this index is similar for the new object and the familiar for *Fmr1*^−/y^ mice (*p* = 0.519; Figure 8A), the total time of objects exploration during this phase was similar between genotypes (*F*_g(1,20)_ = 0.675, *p* = 0.420).

For the NOR2 task, the new object was a white plastic cube so that only the color would discriminate the familiar object from the new object. Once again statistical analysis revealed an impact of the object on the time spent in exploration (*F*_o(1,20)_ = 10.97, *p* = 0.003) and on the number of visits (*F*_o(1,20)_ = 6.01, *p* = 0.023). WT mice were able to distinguish the two objects, as illustrated by a higher exploration time of the new object (*p* = 0.008) and a larger number of nose-contact (*p* = 0.011; Figure 8B). Consequently, WT mice have a significantly higher index for the new object vs. the familiar object (*p* = 0.004). In contrast, *Fmr1*^−/y^ mice explored the two objects equally (time *p* = 0.1133; number *p* = 0.617), similar to the one performed during the familiarization phase. Consequently, discrimination index of the two objects was similar for *Fmr1*^−/y^ mice (*p* = 0.329).

For the NOR3 task, the new object was a black ball so that it differed from the familiar object in its shape but not by color. Once again statistical analysis revealed an impact of the object on the time spent in exploration (*F*_o(1,20)_ = 11.77, *p* = 0.002) and on the number of visits (*F*_o(1,20)_ = 8.32, *p* = 0.009). WT mice have a preference for the new object since they spent more time sniffing it (*p* = 0.002) and came more often into contact with it (*p* = 0.001). The discrimination index indicates that WT mice tended to spend more time in contact with the new object during this choice phase (*p* = 0.075). *Fmr1*^−/y^ mice explored the two objects in a similar way (time *p* = 0.240; number *p* = 1.000), similar to the one performed during the familiarization phase, as illustrated by the discrimination index (*p* = 0.914; Figure 8C). Interestingly, the discrimination index showed that *Fmr1*^−/y^ spend less time exploring the two objects compared to the familiarization phase.

Furthermore, discrimination index of the three versions were statistically different for WT mice ($\chi_{\left(2\right)}^{2}$ = 6.88, *p* = 0.031) but not for *Fmr1*^−/y^ mice ($\chi_{\left(2\right)}^{2}$ = 2.92, *p* = 0.112). More precisely, WT mice exhibited a difference of discrimination index only between the second and the third versions (NOR2–3: *p* = 0.038), and not between other versions (NOR1–2: *p* = 0.213; NOR1–3: *p* = 0.085). This may indicate that color impacts performance more in the NOR test than shape. Besides, it is important to underline that, for each genotype, there was no statistical difference between total durations spent in exploration during choice phases of the 3 versions of the test (WT: $\chi_{\left(2\right)}^{2}$ = 1.42, *p* = 0.489; *Fmr1*^−/y^: $\chi_{\left(2\right)}^{2}$ = 1.29, *p* = 0.231).

Regarding locomotion features, statistical analysis did not underline any difference between genotypes, whatever the phase, for NOR1 (familiarization *F*_g(1,20)_ = 1.591, *p* = 0.221; choice *F*_g(1,20)_ = 0.125, *p* = 0.727), NOR2 (familiarization *F*_g(1,20)_ = 0.010, *p* = 0.920; choice *F*_g(1,20)_ = 0.0004, *p* = 0.983) and NOR3 (familiarization *F*_g(1,20)_ = 0.928, *p* = 0.346; choice *F*_g(1,20)_ = 0.020, *p* = 0.886; Supplementary Figure S2). However, the hyperactivity phenotype of *Fmr1*^−/y^ mice was observed during the first day of habituation phase (*F*_g(1,20)_ = 6.37, *p* = 0.020), where *Fmr1*^−/y^ traveled a higher distance than WT. This difference disappeared in the course of the second day of habituation to the open-field.

Altogether, these results indicate that whatever the parameter modified, color or shape, WT mice were able to distinguish the replaced object, unlike *Fmr1*^−/y^ mice which were not able to discriminate the two objects in any version of the test.

## Discussion

Evaluation of visual function and its effect on visually guided behavior is of high importance to better understand and characterize pathologies associated with visual integration and perception defects. In the FXS, absence of FMRP has been associated to visual system defect in patients, characterized by spatiotemporal visual processing alterations (Kogan et al., 2004b; Farzin et al., 2008) as in its murine model at the retinal level (Rossignol et al., 2014; Perche et al., 2018). However, impact on the visual behavior had never been investigated. Our project aimed to better characterize visual disturbances in *Fmr1*^−/y^ mice from a behavioral perspective.

Contrast perception is a fundamental parameter for functional vision, involved in many tasks and crucial for other visual abilities, such as texture vision (Ginsburg, 2003; Ichihara et al., 2007). In facts, the vision of contrast requires properly measuring a local difference in luminance to detect a target. Contrast perception requires a gain control mechanism occurring in neurons of the early visual system, including retina, lateral geniculate nucleus and V1 cortex (Rathbun et al., 2016). To assess FMRP impact on the vision of contrasts, we implemented the Optomotor Drum test in its standard version (Cowey and Franzini, 1979; Schmucker et al., 2005). Herein, behavioral responses were distinct between WT and *Fmr1*^−/y^ mice. Both genotypes were able to provide a HT in front of the contrasted stimulus, but *Fmr1*^−/y^ mice presented a drop in their response as compared to WT. It is crucial to underline that in this reflex-based test the decrease observed in *Fmr1*^−/y^ mice response might not be due to disinterest or lack of attention, as observed in other behavioral tests (Moon et al., 2006; Casten et al., 2011; Krueger et al., 2011; Kramvis et al., 2013). Indeed, as illustrated by the increased latency to the first HT, *Fmr1*^−/y^ mice showed difficulties in the detection of the target, but when once achieved they followed it in the same way than WT one without variation of HT quality. Therefore, this behavior highlights a clear alteration in *Fmr1*^−/y^ mice abilities to perceive a contrasted stimulus. Interestingly, optomotor response can be affected by alterations of various structures of the visual system. Retina has a clear impact on optokinetic response since Optomotor Drum response is very different between rodents with normal or degenerated retinas (Lawrence et al., 2000; Thaung et al., 2002; Schmucker et al., 2005). Furthermore, the eyes of animals with different retinal states, degenerated or treated, provide different responses to the test (Thomas et al., 2004), clearly indicating that a satisfying retinal perception and transmission, independently to integrative performance, is a crucial parameter for contrast perception. Interestingly, the Ins2Akita/+ mouse model of diabetes with significant retinal alterations, particularly aberrant morphology of ganglion cells neurons characterized by enlarged somas, swollen dendrites and dendritic blebbing, presented an abnormal response to optomotor tasks which can be linked to these retinal neuronal injuries (Akimov and Rentería, 2012). Besides, defects in retinal starburst amacrine cells critically affect the optokinetic response in mice (Yoshida et al., 2001). As *Fmr1*^−/y^ mice exhibit those retinal neuronal defects, and provide electrophysiological evidences (*Bmax* and *n* parameters) that their retinas have an altered contrast sensitivity (Rossignol et al., 2014; Perche et al., 2018), these data likely create a link between the retinal absence of FMRP and *Fmr1*^−/y^ defects in their perception of contrast and motion. Moreover, even if little is known regarding which cerebral structure mediates the optokinetic head tracking in rodents, the SC, which is known to initiate head and eye movement in orienting behaviors (Wurtz and Albano, 1980), may be involved in the optokinetic response. Thus, cellular and functional impairments in the SC of *Fmr1*^−/y^ mice must be also involved in their altered optomotor performance. These observations reinforce the link we hypothesize between the absence of FMRP and contrast and motion perception.

Contrast sensitivity is a crucial property of vision that measures a local difference in luminance necessary to detect a target. In our contrast-shaded Optomotor Drum, contrast may be defined as the luminance difference between the two shades of gray used to compose a pattern, since a dark gray has a weak luminance and a light gray has a high luminance. This definition is similar to the one previously used in study of contrast sensitivity using Optomotor Drum, defining the contrast as the difference in luminance between peak and valley of a sine-wave pattern (Umino and Solessio, 2013). Here, we show that whatever the genotype mice have difficulties to distinguish a sharp contrast in dark conditions. However, as soon as the contrast was somewhat increased WT mice reached their maximal score in contrast detection, whereas *Fmr1*^−/y^ mice did not enhance their response. In this sense, *Fmr1*^−/y^ mice showed a lower sensitivity to contrast, since they demanded a far more increased contrast to enhance their contrast detection ability. A more important leap in contrast is necessary for *Fmr1*^−/y^ to enhance their response towards contrasts. To go further, we can affirm that, in these conditions, we have highlighted an abnormality in the threshold beyond which mice leave their basal response since this threshold is shifted to a higher contrast for *Fmr1*^−/y^ mice compared to WT mice. *Fmr1*^−/y^ mice have a higher contrasted-threshold than WT to convert a weak response in their maximal response. Surprisingly, the *Fmr1*^−/y^ response increase when the contrast was reduced in bright conditions when we could expect it to decrease or stay stable. Even though we remain unable to explain precisely this phenomenon, we can hypothesize that it comes from the difference in visual abilities of rodents between dark and light conditions. As mice are nocturnal mammals, processes that drive their visual abilities are different, depending on the lightness. Herein, this particular result may highlight that, in the *Fmr1*^−/y^ mouse, the visual pathway driving discrimination of bright contrasts is less impacted by the absence of FMRP than visual pathway driving discrimination of dark contrasts. This may explain that performance in a bright contrast condition is better than in middle contrast condition. Therefore, we showed, for the first time, a clear hyposensitivity to contrast in the *Fmr1*^−/y^ mice. Moreover, as mentioned above, mice are nocturnal mammals, and so the perception of contrasts in obscurity is an important element for their survival. As *Fmr1*^−/y^ mice showed defects in perception of contrasts in dark conditions, these mice are more likely to present difficulties in their adaptation to their surrounding environment. This hypo-sensitivity to contrast in dark conditions can be coherently linked to electrophysiological alteration highlighted in the *Fmr1*^−/y^ retina (Rossignol et al., 2014). ERG study in scotopic conditions revealed that *Fmr1*^−/y^ retinas need a higher luminance difference, between the study flash and the background, to initiate a signal transmission in the inner retina, as showed by the *Fmr1*^−/y^y delayed b-wave sensitivity curves. Thus, the contrast needed by the retina to initiate a signal transmission is higher in *Fmr1*^−/y^ mice than in WT. Furthermore, we cannot exclude a brain involvement in such phenotype since a response to contrast is also modulated by the cerebral area of the geniculo-cortical pathway with neurons exhibiting their own gain response to contrast stimulus (Rathbun et al., 2016). Since *Fmr1*^−/y^ mice display molecular and cellular alterations in this pathway, as previously reported, a brain involvement in this contrast sensitivity alteration is highly probable. Once again, these observations reinforce our hypothesis of the link between the absence of FMRP and contrast perception alteration.

Depth perception is an important component of vision enabling three-dimensional visualization of the surrounding environment. It is based on the two-dimensional representation in the retina and requires monocular and binocular inputs (Lashley and Russell, 1934; Walk et al., 1957; DeAngelis et al., 1998). It is also important to note that by determining the depth perception, we evaluate the functional integrity of the retino-geniculo-cortical pathway meaning all the visual axis from the light perception by the retina to the cerebral integration by visual cortex (Fox, 1965; Mazziotti et al., 2017). To understand the role of FMRP on such visual depth perception, the Visual Cliff test was used. As described previously (Baroncelli et al., 2013), wide-type (WT) mice presented an innate tendency to avoid the deep side of the apparatus. However, *Fmr1*^−/y^y mice displayed a clear decrease of the PI for the safe zone, that cannot be attributed to locomotion activity. Therefore, *Fmr1*^−/y^ mice behavior in the Visual Cliff test highlighted a defect in their depth perception. This might affect the hypo-anxiety phenotype obtained with tasks assessing the emotional state of mice, such as the elevated plus maze test as *Fmr1*^−/y^ mice are less able to perceive the depth and, thus, may be less anxious of their environment (Heulens et al., 2012; Chen et al., 2013; Hebert et al., 2014). Interestingly, our results did not show a loss of the preference for the safe zone, but only a decrease in the preference for the safe zone. Thus, *Fmr1*^−/y^ mice are not totally unable to perceive the depth but have a clear impairment in their abilities to distinguish it. We can hypothesize that this phenotype penetrance is linked to the retino-geniculo-cortical pathway state in absence of FMRP. Indeed, it has been clearly demonstrated that modification of this pathway by targeting the retina or the visual brain areas modulate depth perception (Mazziotti et al., 2017; Tzameret et al., 2019). From a molecular standpoint, the decreased efficiency of the retino-geniculo-cortical pathway in *Fmr1*^−/y^ mice must be linked to the impairments of neuron-specific functions such as glutamate/GABA pathways or synaptic transmissions (Davidovic et al., 2011; Doll et al., 2017) due to well described protein deregulation in FXS conditions, such as PSD95, mGlur5, or SNARE complex expression defects (Zhu et al., 2011; Westmark, 2013; Tang et al., 2015; Aloisi et al., 2017). Interestingly, in the Rbfox1 knockout animals characterized by impairments of the visual neuronal circuits due to SNARE complex protein alteration, a similar depth perception defect was observed (Gu et al., 2018). This observation reinforces our data on the direct link between the absence of FMRP and visual depth perception.

For the first time, *Fmr1*^−/y^ mice were investigated with visual specific behavioral tests. These tests brought into light deficits in visual abilities, characterized by an altered perception of perspectives and lower abilities in understanding motion and contrast. Besides, this study highlighted the stability of *Fmr1*^−/y^ mice visual behavioral phenotype over time, particularly from adolescence to late adulthood, similarly to molecular and cellular impairments observed in various visual structures of *Fmr1*^−/y^ mice (Nimchinsky et al., 2001; Perche et al., 2018). Exact origins of defects are difficult to pinpoint since downregulation of *Fmr1* takes place in the retina but also in several brain structures that receive and process visual information. We demonstrated that *Fmr1*^−/y^ mice display clear disturbances that are in their way when perceiving their surrounding environment, even in their usual comfort condition of dim light. Therefore, this pioneering investigation raised the question of the involvement of visual abilities when performing other tasks, and in particular behavioral tests used to assess cognitive skills in mice, and how visual disorders may affect performance.

For a long time, many behavioral scientists argued that laboratory mice were nearly blind, or at least did not use vision in behavioral tasks (Baker, 2013). Recently, some studies have shown that vision is important in mice, as exampled by the accurate capture of prey carried out not only through defensive behaviors (Hoy et al., 2016). Furthermore, many visual-spatial behavioral tasks require the ability to detect and distinguish several clues placed in the experiment room in order to establish a mental map of the room. Thus, an impaired vision can affect mice performance in many behavioral tasks. Cognitive traits of *Fmr1*^−/y^ mice had been investigated with behavioral tasks involving vision of objects or clues. Since our study shows that these mice display abnormalities in visual perception, we investigated the link between visual perception and the achievement of a cognitive task commonly used in laboratories, the NOR test. However, since *Fmr1*^−/y^ mice present an impaired recognition memory, as widely described in the literature (Ventura et al., 2004; Pardo et al., 2017; Yan et al., 2018; Yau et al., 2018), the study of the involvement of vision in this type of test can only be based on the behavior of WT mice.

We performed the NOR in its standard version (NOR1), in which the novel object presented in phase 3 differed from the familiar by its shape and its color (but has the same texture), and then in a modified task, in which the new object differed only by its color or its shape, in order to establish whether these characteristics could contribute to the recognition of the presented object. With the standard version, we obtained results similar to the literature (Bhattacharya et al., 2012; King and Jope, 2013; Gomis-González et al., 2016; Costa et al., 2018), with WT mice showing a preference for the novel object. Furthermore, it appears that whatever the modified feature (color or shape) WT mice were able to distinguish the familiar object from the new one, in the same way that in the NOR1. These results in WT mice indicate that discrimination of two objects is impacted by both color and shape. Furthermore, the comparison of discrimination indexes allowed us to highlight that color seemed a predominant characteristic in the process of visual recognition. Therefore, results we obtained with the two modified versions of the test showed that, beyond to the cognitive aspect, this test involves visual abilities.

In this type of behavioral task, an interaction between the cognitive disorders and visual impairment may exist. Herein, *Fmr1*^−/y^ mice present difficulties in distinguishing an unknown object from a familiar object in all versions of the test, as described in the literature with the standard NOR (Bhattacharya et al., 2012; King and Jope, 2013; Gomis-González et al., 2016; Costa et al., 2018). The previously demonstrated visual impairments in *Fmr1*^−/y^ mice may partly explain this phenotype. Since cognitive defects of the *Fmr1*^−/y^ mouse strain is well-described and knowing that these mice present visual impairments, we cannot ignore that visual alterations must affect their NOR performance. Previously, some misleading cognitive studies have been questioned because they did not sufficiently take into account visual disturbances. As an example, the abnormal behavioral response of a mouse model with Huntington’s Disease (R6/2) to Morris water maze and Visual Cliff tests had been linked to cognitive impairments known in this pathology (Lione et al., 1999). Nevertheless, eventually researchers discovered that the mutation in mice also leads in to a retinal degenerative anomaly (Ragauskas et al., 2014), and thus affected their behavioral responses. This study clearly demonstrated that it is crucial to first investigate mice visual abilities before performing such behavioral tasks. Therefore, our results underline that it becomes important to consider visual modality disorders when performing various behavioral tests, not only regarding the *Fmr1*^−/y^ mouse strain but more broadly for many rodent models of neuropsychiatric pathologies.

Neurosensorial abnormalities are a strong phenotype of the FXS conditions. Indeed, odorant sensitivity (Schilit Nitenson et al., 2015) and nociceptive responses after a local acute inflammation (Price et al., 2007; Busquets-Garcia et al., 2013) are significantly lower in the FXS model whereas an excessive excitability of auditory processing (Garcia-Pino et al., 2017) and an exaggerated response to whisker stimulation (He et al., 2017) were observed. Regarding vision, few data were reported and they were associated to cognitive mechanism such as the *Fmr1*^−/y^ delayed learning on visual discrimination tasks (Goel et al., 2018). Herein, we clearly highlighted specific visual acuity skills defect. It’s interesting to note that an electrophysiological study of *Fmr1*^−/y^ mice retina had shown an hypersensitivity to visual stimuli, in the sense that they provided an exacerbated transmission in the inner retina, together with an altered sensitivity toward contrasts (Rossignol et al., 2014; Perche et al., 2018). Our behavioral results, rather, provided a hyposensitivity to visual stimuli, since *Fmr1*^−/y^ mice were less sensitive to perspective, motion and contrast than WT littermates.

Interestingly, this visual phenotype observed in *Fmr1*^−/y^ mice is reminiscent of visual abnormalities described in FXS patients. Indeed, from childhood patients exhibit pervasive impairments in motion perception (Kogan et al., 2004b), together with deficits in the detection of moving contrasted stimuli (Farzin et al., 2008). Significant impairments have been observed in FXS patients while undergoing visual tests involving dynamics contrasted-or-textured stimuli and are in agreement with the phenotype described herein using the Optomotor Drum test. Furthermore, our present study described a deficit in perspective perception in the absence of FMRP, but to date, no data is available regarding the specific investigation of this visual trait in FXS patients. Yet, considering our present results, it would be pertinent to address how FXS patients perceive depth and perspective. This skill, together with contrast and motion perceptions, comprised visual abilities which are crucial to development and in performing common tasks, such as locomotion control, gait, orientation and obstacles position planning (Jahn et al., 2001; Rietdyk and Rhea, 2006; Hallemans et al., 2009, 2010). Thus, altered visual skills in FXS patients could contribute to their delayed sensori-motor features (Baranek et al., 2005) and to their impairments in neuropsychological tasks that require drawing skills and fine psychomotor coordination (Crowe and Hay, 1990; Freund and Reiss, 1991; Cornish et al., 1999). Although these tasks are multifactorial and performance is affected by many causes, visual-motor abilities are a common feature and largely affect patients’ performance when altered. More importantly, the deficit showed by FXS patients in tasks assessing emotion recognition on faces may reflect information processing and memory deficits rather than dysfunction in emotion-recognition (Turk and Cornish, 1998). Various studies related deficits in scanning path and gaze when patients analyzed a face, with a reduced attention given to the eyes (Dalton et al., 2008; Shaw and Porter, 2013) rather than an absence of recognition or reaction toward an emotion. Decoding facial characteristics, and even more an emotion, is a complex task beginning with the perception of sharp and discrete clues, as slight shadows, folding, and modifications in facial texture. Our study highlighting deficits in contrast, motion and perspective perception in the absence of FMRP, together with previous investigation of visual skills in patients, theorizes that alteration in facial analyses is due to an unclear and unstable perception of face zones and of delicate contrasted and textures clues displayed by an emotion on a face.

In conclusion, our study filled the gap in the sensory investigation of *Fmr1*^−/y^ mice, and reinforces the idea we previously put forward (Perche et al., 2018): *Fmr1*^−/y^ mice exhibit a complex sensorial spectrum which should be called “dys-sensitivity.” Interestingly, this visual phenotype observed in *Fmr1*^−/y^ mice is similar of visual abnormalities described in FXS patients (Kogan et al., 2004a; Farzin et al., 2008). Our results strengthen clinicians’ theories assuming that sensory anomalies in FXS, or in other neuropsychiatric disorders as Autism Spectrum Disorder (ASD), lead to an incapacity for patients to understand and interact with their environment, explaining behavioral abnormalities. Moreover, our study underlines the significance of visual behavior alterations in FXS conditions and how relevant it is to assess visual skills in neuropsychiatric models before performing behavioral tasks, such as cognitive assessments.

## Data Availability Statement

The datasets generated for this study are available on request to the corresponding author.

## Ethics Statement

All experimental protocols received full review and approval by the regional animal care and use committee (Comité Régional d’Ethique à l’Expérimentation Animale—CREEA—TSA-DM Therapie1100) prior to conducting the experiments.

## Author Contributions

CF, BH and OP conceived and designed the experiments. CF, BH, GM-D and KP-M performed the behavioral experiments. CF and BH analyzed the data. CF, BH and OP wrote the manuscript. CF, BH, MA, AM, J-CB, JP, SB and OP edited the manuscript. All authors read and approved the final manuscript.

## Conflict of Interest

J-CB is employed by company Key-Obs. The remaining authors declare that the research was conducted in the absence of any commercial or financial relationships that could be construed as a potential conflict of interest.

## Abbreviations

FXS: Fragile X Syndrome
ID: Intellectual Disability
ASD: Autism Spectrum Disorder
FMRP: Fragile Mental Retardation Protein
FMR1: Fragile Mental Retardation 1
ERG: Electroretinogram
WT: Wild-type
NOR: Novel Object Recognition
PI: Preference Index
HT: ead-tracking
ITI: Intertrial interval
RGB: Red Green Blue color value.
