# Supplementary material for: Visual Behavior Impairments as an Aberrant Sensory Processing in the Mouse Model of Fragile X Syndrome
Source: Front Behav Neurosci. 2019 Oct 2;13:228. doi: 10.3389/fnbeh.2019.00228 (PMC6797836; doi:10.3389/fnbeh.2019.00228)
Supplement: Supplementary file 1 [file Data_Sheet_1.docx]

**Supplementary Data**

**Supplementary Material: Optomotor Drum test**

In order to check that results obtained were not due to a laterality disorder, the analysis was performed for each rotation direction (Figure S3). Statistical analysis did not underline significant interaction between age and genotype for number of HT of both rotation senses [clockwise: F_a,g_(2,80) = 0.123, *p* = 0.8846; counterclockwise: F_a,g_(2,80) = 0.1325, *p* = 0.8761] as well as for time spent in HT [clockwise: F_a,g_(2,80) = 0.3605, *p* = 0.6985; F_a,g_(2,80) = 0.0246, *p* = 0.9757]. Similarly, we did not record any significant difference in mean duration of a HT between ages for WT mice [χ^2^(2) = 0.023, *p* = 0.988] nor for *Fmr1^-/y^* ones [χ^2^(2) = 5.122, *p* = 0.077].

At 1 month old, a significant genotype effect was noticed on number of HT when the drum was clockwise rotating [F_g_(1,80) = 63.099, *p* < 0.0001] as when it was counterclockwise rotating [F_g_(1,80) = 42.5204, *p* < 0.0001]. Indeed *Fmr1^-/y^* mice provided a significant decrease in its number of HT in both senses of rotation (clockwise *p* < 0.0001; counterclockwise *p* = 0.0006) (Figure S3A). Similarly, a significant genotype effect was got on the time spent in HT when the drum was clockwise rotating [F_g_(1,80) = 33.048, *p* < 0.0001] as when it was counterclockwise rotating [F_g_(1,80) = 16.538, *p* = 0.0001] (Figure S3B). However, WT and *Fmr1^-/y^* mice did not show a significant difference in their mean duration of a HT when rotation was clockwise (*p* = 0.1824) as when it was counterclockwise (*p* = 0.9157) (Figure S3C). Three- and 6-months old mice provided similar results profiles in terms of number (Figure S3A), time (Figure S3B) and mean duration of HT (Figure S3C). In clockwise rotation, whatever the age, *Fmr1^-/y^* mice provided a significantly decreased number of HT (3 months old: *p* < 0.0001; 6 months old: *p* < 0.0001), and time spent in HT (3 months: *p* = 0.0079; 6 months old: *p* =0.0016) in comparison with WT mice (Figure S3A and B). WT and *Fmr1^-/y^* mice did not provide any significant difference in mean duration of HT at 3 months old (*p* = 0.2688) and 6 months old (*p* = 0.3462) (Figure S3C). Similarly, in counterclockwise rotation, whatever the age, *Fmr1^-/y^* mice provided a significantly decreased number of HT (3 months old: *p* = 0.0006; 6 months old: *p* < 0.0001), and time spent in HT (3 months: *p* = 0.0284; 6 months old: *p* =0.0265) in comparison with WT mice (Figure S3A and B). WT and *Fmr1^-/y^* mice did not provide any significant difference in mean duration of HT at 3 months old (*p* = 0.1019) and 6 months old (*p* = 0.1414) (Figure S3C).

Whatever the rotation direction, *Fmr1^-/y^* mice provided a response to the Optomotor Drum test decreased by approximately 35% when compared to WT mice response, from 1 to 6 months old. Thus, the decrease in response provided by *Fmr1^-/y^* mice in the total test occurred regularly during both phases of the test (clockwise and counterclockwise).

**Supplementary Figures**


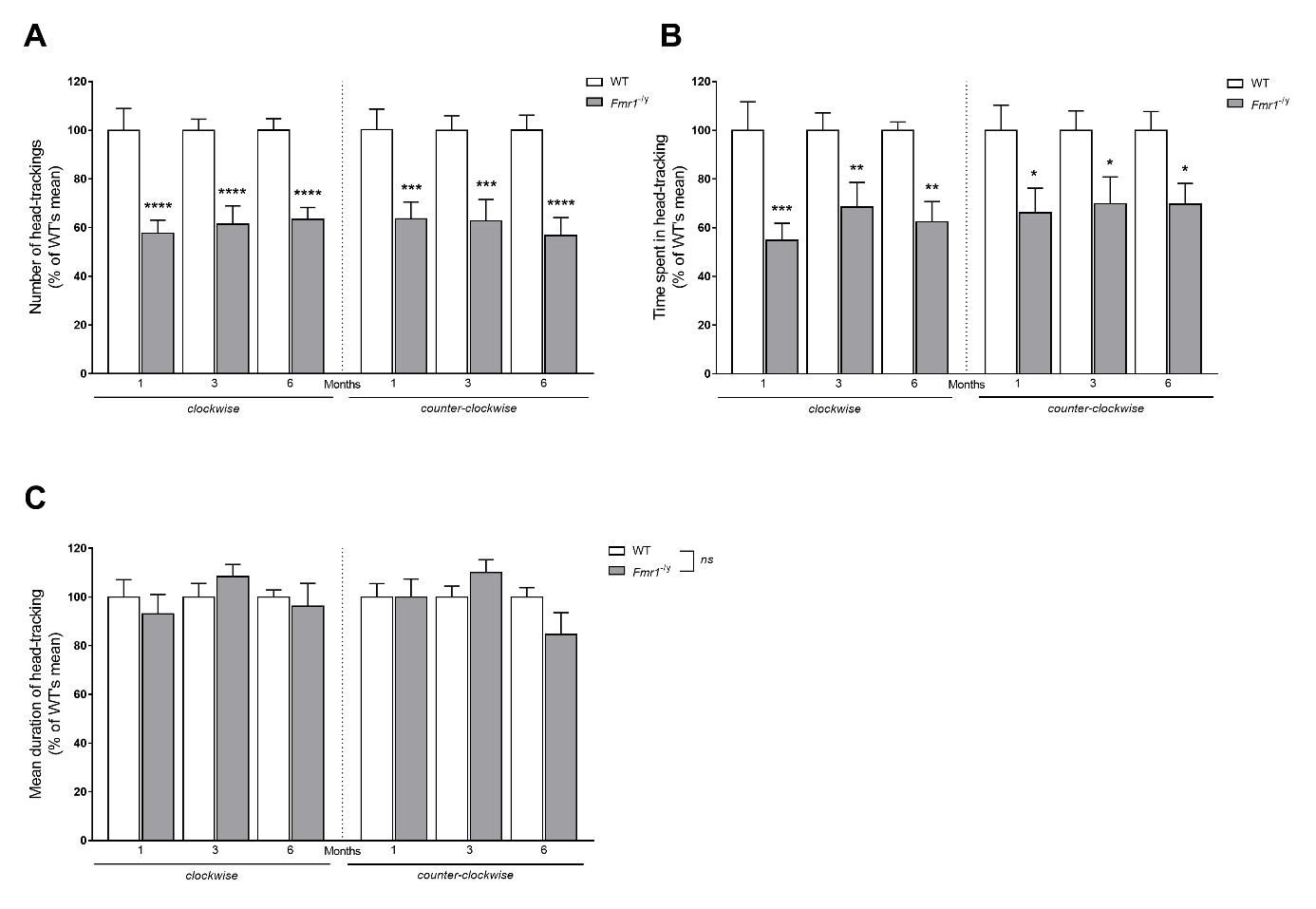


**Figure S1**

**Optomotor Drum test.** Histograms represent parameters scored during clockwise and counter-clockwise phases of the Optomotor Drum test, namely (**A**) total number of head-trackings (HT), (**B**) total time spent in HT, hence (**C**) mean duration of one HT, at each age tested (1 month: WT *n*=12 ; *Fmr1^-/y^* *n* = 18 ; 3 months: WT *n* = 15 ; *Fmr1^-/y^* *n* = 13 ; 6 months: WT *n* = 14 ; *Fmr1^-/y^* *n* = 14). Total durations and mean durations scored in seconds. Numbers, total durations and mean durations expressed in % of results got with the WT group at the corresponding age (WT littermate). Data represent mean ± SEM. Significant differences between WT and *Fmr1^-/y^* are noted by **p* < 0.05; ***p* < 0.01; ****p* < 0.001; *****p* < 0.0001.


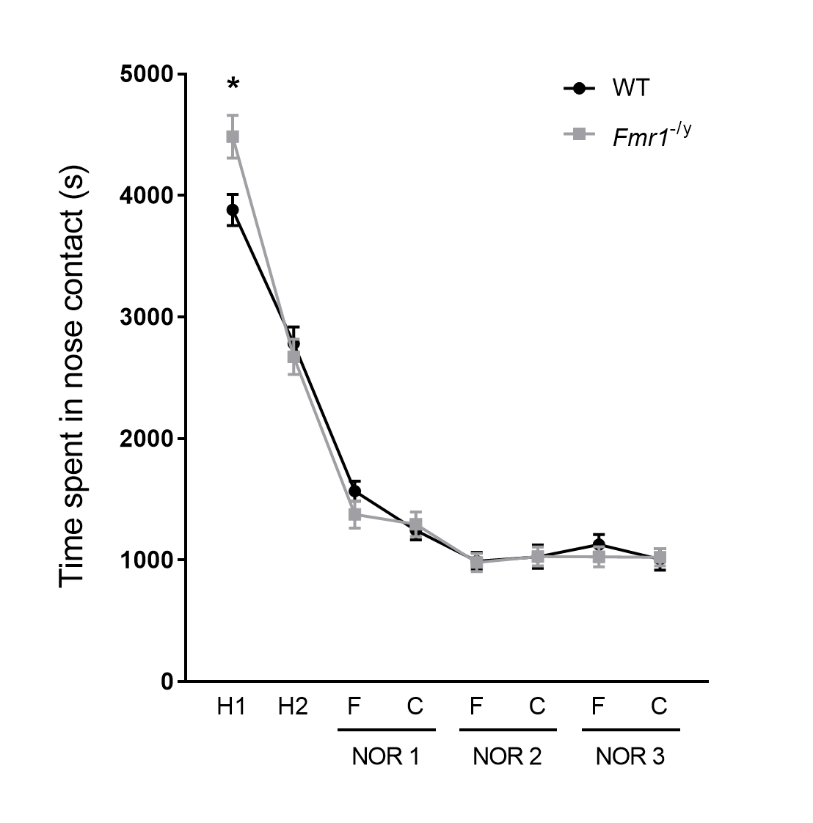


**Figure S2**

**Locomotion parameters in Novel Object Recognition (NOR).** Locomotion was assessed thanks to total distances moved during the different phase of the three version of the NOR, including the two sessions of habituation (WT *n* = 9, *Fmr1^-/y^* *n* = 13). H1: day 1 of open-field habituation; H2: day 2 of open-field habituation; F: familiarization phase; C: choice phase. Distance scored in cm. Data represent mean ± SEM. Significant differences between WT and *Fmr1^-/y^* are noted by **p* < 0.05.
